# Current problematic and emergence of carbapenemase-producing bacteria: a brief report from a Libyan hospital

**DOI:** 10.11604/pamj.2017.26.180.9637

**Published:** 2017-03-29

**Authors:** Asma Elramalli, Nariman Almshawt, Mohamed Omar Ahmed

**Affiliations:** 1 Tripoli Medical Centre, Tripoli, Libya; 2 Department of Microbiology and Parasitology, Faculty of Veterinary Medicine, University of Tripoli, Libya

**Keywords:** Carbapenemase-producing bacteria, antimicrobial resistance, public health, Libya

## Abstract

A collection of 94 Gram-negative bacteria isolates, showing different antimicrobial resistance phenotypes including to the carbapenem classes was investigated. Strains were originated form clinical sources from a single hospital in Tripoli, Libya during 2015 and were identified based on cultural and phenotypic characteristics, and fully characterized by the VITEK automated system. Forty-eight percent (48%) of the collection was identified as Acinetobacter baumannii, 50% Klebsiella pneumoniae and 2% Escherichia coli. Resistance to the carbapenem classes was reported in 96% of the A. baumannii strains and 94% of the K. pneumonia strains. Seventy-eight percent (78%) of the isolates showed different multidrug-resistant (MDR) phenotypes, of which K. pneumoniae expressing the highest rates of MDRs(i.e. 91%). Emergence of resistance to carbapenems in the Gram-negative bacteria is a challenging global problem, particularly for Africa. Surveillance of these pathogens and appropriate actions are urgently required in Libyan healthcare settings.

## Introduction

The emergence of antimicrobial resistance (AMR) is a major threat to human health and is occurring at an alarming rate throughout the globe, diminishing the already limited therapeutic options [1]. Low-income regions, such as Africa, are at high and particular risk. Such threats are complicated by the underdeveloped regional conditions and socioeconomic factors that are associated with healthcare settings and community-acquired pathogens [2]. AMR in Africa is a rising problem and frequently reported from clinical, non-clinical and environmental sources [2, 3].

Carbapenems are potent 46;-lactam antibiotics that are considered as the last resort option for treating serious nosocomial infections caused by a broad spectrum of Gram-negative bacteria [4]. Prior to 2000, only few clinical isolates showed resistance to carbapenems, mostly represented by Acinetobacter baumannii and Pseudomonas aeruginosa. Thereafter, carbapenem resistance has emerged as a major, global health concern and a major clinical problem [5]. Carbapenemase-producing bacteria has come to the forefront as a global concern representing a serious medical and public health issue due to multidrug resistances (MDRs) that involve valuable therapeutic choices, including the so-called drugs of last resort [6-9].

Over the past decade, studies and investigations have reported the problem of AMR in Libya among different nosocomial bacterial, particularly methicillin-resistant *Staphylococcus aureus* (MRSA) [10, 11]. Recent and increasing reports have also documented the carbapenamase-producing bacteria, mainly involving Libyan traveller patients [6, 12-16]. These recent and limited studies have reported different carbapenemase-encoding genes in the Gram-negative bacteria, including the blaVIM-2, blaNDM and OXA-genes [17, 18]. Nevertheless, surveillance and epidemiological investigations on AMR bacterial pathogens in this particular region remain inadequate, limited and incomplete. In this short report, we investigate and provide data on the species spectrum and the antibiotic susceptibility patterns of 94 Gram-negative bacteria that were collected in 2015 from clinical cases at a single Libyan hospital in Tripoli. These strains were isolated and collected on the basis of the expressed resistance to the carbapenem classes. This brief work aimed to report and discuss the current threat and future concern of carbapenemase-producing Gram-negative bacteria in Libyan hospitals.

## Methods

The 94 Gram-negative bacterial strains were initially isolated and identified based on cultural and biochemical characteristics (i.e. Gram stain, catalase reactivity, and API 20E system). Isolates were defined and characterized at species level and the antibiotic susceptibility profile was determined using the VITEK automated system (VITEK-Compact 2). The strains were selected and collected based on the expressed resistance to at least one carbapenem class (i.e. meropenem or/and ertapenem) and stored at -20^o^C. The VITEK system tested *A. baumannii* only against meropenem, while the *E. coli* and *K. pneumoniae* were tested against both of the carbapenem classes. The Multidrug resistance phenotypes (MDR) (i.e. based on the expressed resistance to at least 5 different antimicrobial classes) were also characterized.

## Results

As a result, 45/94 (48%) were *A. baumannii*, 47/94 (50%) were *K. pneumoniae* and only 2/94 (2%) were *E. coli*. Of the *A. baumannii* strains, 43/45 (96%) showed resistance to meropenem. Of the *K. pneumonia* strains, 33/47 (70%) showed resistance to meropenem and 44/47 (94%) showed resistance to ertapenem (Table 1). Both *E. coli* isolates show resistance to ertapenem and only one strain show resistance to meropenem. A total of 73/94 (78%) of the strains showed different MDR phenotypes; 67% of *A. baumannii* and 91% of *K. pneumoniae* strains.

**Table1:** Antimicrobial resistance of the collection-strains

Antimicrobial agents	Proportion of strains (%)	
	Klebsiella pneumoniae	Acinetobacter baumannii
AMP	100	100
AMS	100	96
TZP	100	96
CZ	100	100
FOX	100	100
CAZ	100	93
CRO	100	100
FEP	70	96
ETP	94	--
MEM	70	96
AMK	2	--
GEN	98	53
TOB	98	27
CIP	94	94
LEV	94	56
NIT	98	100
SXT	45	44

**None; AMP**, Ampicillin; **AMS**, Ampicillin/Sulbactam; **TZP**, Pipracillin Tazobactam; **CZ**, Cefazolin; **FOX**, Cefoxitin; **CAZ**, Ceftazidime; **CRO**, Ceftriaxone; **FEP**, cefepime; **ETP**, Ertapenem; **MEM**, Meropenem; **AMK**, Amikacin; **GEN**, gentamycin; **TOB**, Tobramycin; **CIP**, Ciprofloxacin; **LVX**, levofloxacin; **NIT**, Nitroforantion; **SXT**; Trimethoprim/sulfamethoxazole: co-trimoxazole,

## Discussion

*Staphylococcus aureus, Pseudomonas spp.* and *Klebsiella spp*. were previously reported as the most frequent causes of nosocomial infections, highlighting the reported emerging challenge of antimicrobial resistance in Libyan hospitals [10]. A recent investigation involving a single Libyan hospital has found *P. aeruginosa*, *A. baumannii* and *S. aureus* as the most identified nosocomial bacterial strains expressing high rates of MDR phenotypes, including to the cephalosporins and carbapenems (personal unpublished 2015 data). In the current brief report, *A. baumannii* and *K. pneumonia* were identified as the dominant bacteria expressing high level of resistance to different carbapenems classes . The *Klebsiella* strains showed a remarkable rate of MDRs and different antimicrobial resistance patterns (Table 1). Previously, the extended spectrum beta-lactamases (ESBLs)- in the Gram-negative isolates from Libya hospitals was reported at a range of 9-15% [10].. Generally, MDR and ESBLs are frequent among the clinical strains of Gram-negative rods of *P. aeruginosa*, *A. baumannii and K. pneumonia*, and show the concomitant resistance phenotypes [3, 13, 19].

The carbapenemase-producing bacteria, are generally recognized as difficult to identify and estimate [9]. The current estimates of carbapenemase-producing bacteria for Africa range from 2.3% to 67.7% [8]. The North African and Mediterranean regions have recently been designated as an endemic/reservoir area of the carbapenemase-producing bacteria (i.e. OXA-48 type) [9, 6, 7, 14]. Globally, KPC, VIM, IMP, NDM, and OXA-48 are the most prevalent β-lactamase classes produced by the carbapenemase-producing bacteria [9].

The documented cases of carbapenemase-producing bacteria from Libya have exclusively involved Libyan travellers and described MDR phenotypes in patients with high co-colonization rates and a pre-hospitalization history in Libyan healthcare settings [12-16]. These reports have documented MDR in *K. pneumonia* and *A. baumannii* strains harbouring either or a combination of OXA-48, OXA-23 and NDM-1. Other report have also demonstrated the presence of *bla_VIM-2_* gene in *P. aeruginosa* and OXA-carbapenemase-encoding genes (i.e. *bla_OXA-23_*, bla_OXA-24_- and bla_OXA-48_) in *A. baumannii* isolates from hospitalized patients in a Libyan hospital [17].

The carbapenemase-producing bacteria, are generally recognized as difficult to identify and estimate [9]. The current estimates of carbapenemase-producing bacteria for Africa range from 2.3% to 67.7% [8]. The North African and Mediterranean regions have recently been designated as an endemic/reservoir area of the carbapenemase-producing bacteria (i.e. OXA-48 type) [6, 7, 9, 14]. Globally KPC VIM IMP NDM and OXA-48 are the most prevalent β-lactamase classes produced by the carbapenemase-producing bacteria [9]. The carbapenem-resistant bacteria in the Gram negative bacteria are a global emergent threat and surveillance and monitoring studies are urgently needed in Libya hospitals. Unfortunately, due to limited financial resources, investigating ESBL genes within the current collection was not possible. Urgent actions and investigations are extremely required to evaluate and understand the extent of carbapenemase-Gram-negative bacteria in Libyan health care settings.

## Conclusion

The antimicrobial resistance (AMR) pose a serious medical and public health concern. The recent published reports and the current information reveal a current and prospective concern. The reporting and rising of AMR in Libya has been mainly attributed to the consumption of antibiotic drugs (i.e. β-lactam drugs) and to the improved identification methods and laboratory skills. However, epidemiological data and molecular investigations remain inadequate from healthcare settings and absent from the community. Clearly, lack of epidemiological and molecular investigations, inadequate surveillance and monitoring studies, complicated socioeconomic factors and underdeveloped healthcare infrastructures are major factors contributing in the development and spreading of AMR. Professional development of healthcare personnel, educational campaigns to increase awareness of AMR and the need for rational use of antibiotics are also important steps to control the spread of AMR

### What is known about this topic

- Carbapenemase-producing bacteria is an emerging global problem especially for the developing regions, particularly for Africa;
- It is already reported from different regions including from the North African countries.
- These resistant strains were also reported from Libyan hospitals and travelled patients however limited attention and information are currently available.

### What this study adds

- This short report reveal the importance to focus on such emergent problem and the associated concern on antimicrobial based therapies particularly for Africa.
- This short paper provides useful information on the susceptibility patters of clinical isolates and can aid clinicians in providing the suitable therapies;
- This brief report can also help epidemiologist and clinical bacteriologist to understand the serious concern of carbapenemase-producers and the extent of the problem within the Libya health care system and take appropriate actions.

## References

[1] World Health Organization (2014). Antimicrobial resistance: global report on surveillance.

[2] (2013). WHO-Antimicrobial resistance in the African Region: Issues, challenges and actions proposed Key determinants for health in the African Region African Health Monitor.

[3] Papp-Wallace KM, Endimiani A, Taracila MA, Bonomo RA (2011). Carbapenems: past, present and future. Antimicrob Agents Chemother.

[4] Uwingabiye J, Frikh M, Lemnouer A, Bssaibis F, Belefquih B, Maleb A, Dahraoui S, Belyamani L, Bait A, Haimeur C, Louzi L, Ibrahmi A, Elouennass M (2016). Acinetobacter infections prevalence and frequency of the antibiotics resistance: comparative study of intensive care units versus other hospital units. Pan Afr Med J.

[5] Zavascki AP, Carvalhaes CG, Picao RC, Gales AC (2010). Multidrug-resistant Pseudomonas aeruginosa and Acinetobacter baumannii: resistance mechanisms and implications for therapy. Anti-Infect Ther.

[6] Djahmi N, Dunyach-Remy C, Pantel A, Dekhil M, Sotto A, Lavigne J (2014). Epidemiology of carbapenemase-producing Enterobacteriaceae and Acinetobacter baumannii in Mediterranean countries. Biomed Res Int.

[7] Bakour S, Olaitan AO, Ammari H, Touati A, Saoudi S, Saoudi K, Rolain JM (2015). Emergence of Colistin- and Carbapenem-Resistant Acinetobacter baumannii ST2 Clinical Isolate in Algeria: First Case Report. Microb Drug Resist.

[8] Manenzhe RI, Zar HJ, Nicol MP, Kaba M (2015). The spread of carbapenemase-producing bacteria in Africa: a systematic review. J Antimicrob Chemother.

[9] Nordmann P, Naas T, Poirel L (2011). Global spread of Carbapenemase-producing Enterobacteriaceae. Emerg Infect Dis.

[10] Ghenghesh KS, Rahouma A, Tawil K, Zorgani A, Franka E (2013). Antimicrobial resistance in Libya: 1970-2011. Libyan J Med.

[11] Abujnah AA, Zorgani A, Sabri MA, El-Mohammady H, Khalek RA, Ghenghesh KS (2015). Multidrug resistance and extended-spectrum beta-lactamases genes among Escherichia coli from patients with urinary tract infections in Northwestern Libya. Libyan J Med.

[12] Kaase M, Pfennigwerth N, Szabados F, Gatermann S OXA-48, OXA-23 and NDM-1 carbapenemases in Gram-negative bacteria from patients from Libya. "the Twenty-Second European Congress of Clinical Microbiology and Infectious Diseases, London" (ECCMID), London, UK, 20 2014 May 13;10:305784.

[13] Pirs M, Andlovic A, Cerar T, zohar-cretnik T, Kobola L, Kolman J, Frelih T, Presern-Strukelj M, Ruzic-Sabljic E, Seme K (2011). A case of OXA-48 carbapenemase-producing Klebsiella pneumoniae in a patient transferred to Slovenia from Libya, November 2011. Euro Surveill.

[14] Ben Nasr A, Decré D, Compain F, Genel N, Barguellil F, Arlet G (2013). Emergence of NDM-1 in association with OXA-48 in Klebsiella pneumoniae from Tunisia. Antimicrob Agents Chemother.

[15] Hammerum AM, Larsen AR, Hansen F, Justesen US, Friis-Møller A, Lemming LE, Fuursted K, Littauer P, Schønning K, Gahrn-Hansen B, Ellermann-Eriksen S, Kristensen B (2012). Patients transferred from Libya to Denmark carried OXA-48-producing Klebsiella pneumoniae, NDM-1-producing Acinetobacter baumannii and meticillin-resistant Staphylococcus aureus. Int J Antimicrob Agents.

[16] Zorgani A, Ziglam H (2013). Injured Libyan combatant patients: both vectors and victims of multiresistance bacteria. Libyan J Med.

[17] Mathlouthi N, Areig Z, Al Bayssari C, Bakour S, Ali El Salabi A, Ben Gwierif S, Zorgani AA, Ben Slama K, Chouchani C, Rolain JM (2015). Emergence of Carbapenem-Resistant Pseudomonas aeruginosa and Acinetobacter baumannii Clinical Isolates Collected from Some Libyan Hospitals. Microb Drug Resist.

[18] Kraiem AG, Zorgani A, Elahmer O, Hammami A, Chaaben BM, Ghenghesh KS (2015). New Delhi metallo-β-lactamase and OXA-48 carbapenemases in Gram-negative bacilli isolates in Libya. Libyan J Med.

[19] Potron A, Poirel L, Nordmann P (2015). Emerging broad-spectrum resistance in Pseudomonas aeruginosa and Acinetobacter baumannii: Mechanisms and epidemiology. Int J Antimicrob Agents.

